# Parliament and Publications

**Published:** 1910-08-13

**Authors:** 


### Parliament and Publications.

### LUNACY IN ENGLAND AND WALES.

Valuable information relative to lunacy in England and Wales is contained in the sixty-fourth report of the Commissioners in Lunacy which has just been published. On January 1, 1910, the number of certified insane people under care in England and Wales was 130,553, a number exceeding that recorded on January 1, 1909, by 1,766. This increase for 1909 may be contrasted with that of 2,703 for 1908, of 2,096 for 1907, and 2,009 for 1906. The average annual increase for the ten years ending December 31, 1909, was 2,394; and that for the five years ending on the same date, 2,145. The increase for the year 1909 was, therefore, 628 below that of the annual average of the decennium, and 379 below that for the quinquennial period. It is lower than any such yearly increase since 1901, and 243 less than that of the smallest increment in the other years of the decennium—namely, 2,009 in 1906.

Compared with the figures of last year the numbers resident in the county and borough asylums have increased by 1,653, those in registered hospitals by 23, in the metropolitan licensed houses by 52, and the State criminal asylums of Broadmoor and Parkhurst by 11. There has also been an increase of 36 in the numbers under care as single private patients, and of those living with friends in the receipt of outdoor relief of 153, the former having risen in number by 6.4 per cent., and the latter by 2.7 per cent. On the other hand, those in provincial licensed houses have decreased by 30, in the naval and military hospitals by 4, in ordinary workhouses by 31, and in the metropolitan district asylums by 97. Of the totals in residence the inmates of county and borough asylums increased by 1.7 per cent., of metropolitan licensed houses by 3.1 per cent., of registered hospitals by 0.5 per cent.; and the decrease in provincial licensed houses amounted to 2.2 per cent., in the metropolitan district asylums to 1.4 per cent., and in ordinary workhouses to 0.3 per cent.

The private patients under care on January 1, 1910, numbered 10,616 (males 4,622, females 5,994), an increase on the figures of the preceding year of 223, or 2.1 per cent. The pauper patients under care on January 1, 1910, were 118,901 (males 55,121, females 63,780), amounting, as last year, to 91 per cent. of all the certified insane. The average annual increase for this class of patients during the last ten years was 2,187, so that the figures for 1910 are as much as 663 below that mean, and 864 below the increase for 1908. The rate of increase was 1.3 per cent. on last year's numbers, being thus below the rate in the case of private patients. This rate for males was nearly 1.4, and for females 1.2 per cent. The rise in the numbers of insane outdoor paupers is quite an exceptional feature, since for several years their number had been falling off. In the county and borough asylums there were 1,473 more pauper patients in residence at the beginning of this year than on January 1, 1909, an increase of nearly 1.6 per cent. The criminal insane (males 785, females 251) show a net increase of 19.

The report contains some interesting statistics showing the increase in insanity during the past fifty-one years. On January 1, 1859, there were known to be under care 36,762 persons certified as insane, and on January 1, 1910, the number so notified amounted to 130,553, an increase on the figures of 1859 of 255.1 per cent. During the same period the estimated population of England and Wales had increased by 93.7 per cent. Whilst the general population is estimated to grow by fairly regular increments year by year, the numbers of the insane have increased less steadily. It is worthy of note that although the estimated population in 1910 is 12.2 per cent. above that in 1900, and that of the number of insane 22.4 per cent., yet, in the rate of increase during 1909 there is a marked approximation between the two series—that of the general population being reckoned to be 1.16 per cent., and that of the insane 1.37 per cent. If, therefore, the rate of increase of the insane should continue to fall, it may shortly come down to the same level as that of the general community. The private patients, who in 1900 numbered 8,813, have increased by 1,910 to 10,616; their ratio to population, which was 2.73 in the former, being 2.93 in the latter, being the highest in the period 1859 to 1910, except in 1879, when it was 2.97. The pauper ratios, on the other hand, have risen continuously from 15.95 in 1859 to 32.87 in 1910. The ratio for all insane was 18.67 per 10,000 in 1859, so that it has increased by 93.3 per cent. in the fifty-one years.

The number of patients under detention on January 1, 1909, in all institutions (exclusive of idiot establishments) and in private single care was 102,908, being an increase of 28.9 per cent. on the number similarly detained on January 1, 1899—namely, 79,833. At the close of the year there remained under detention 104,607. Of those discharged, 7,831 are stated to have recovered, and of them, 7,108, or 90.7 per cent., were discharged from the county and borough asylums. The recovery rate, reckoned upon the total admissions, was 35.98, being 0.6 higher than the rate for 1908 and 0.92 below the average for the ten years 1900 to 1909 inclusive. The rate for females (35.53) was higher than that for males (32.13), the former being 1.79 above and the latter 0.68 below the rate for 1908. Those who were absolutely discharged as "relieved" or "not recovered"—namely 2,186—were almost precisely 10 per cent. of the total admissions. The deaths numbered 10,138, exceeding the number for 1908 by 446. The death rate reckoned on the daily average number resident was 9.78 per cent., being 0.18 below the average for the ten years. The rate for males was 10.97 and that for females 8.75.

This death rate is more than six times as high as the general death rate. Exclusive of idiot establishments, the ratio of deaths in all institutions for the insane per 1,000 living was, for males 105.2, for females 84.9. Amongst the general population in the same year this ratio was 15.7 and 13.7. The contrast in the two series in respect to mortality rates is more marked in earlier than later age periods. For instance, at ages twenty to twenty-four the death rate amongst the insane community was for males more than fifteen and for females nearly twenty times as high as that in the general population at the same ages; at ages thirty-five to forty-four it was ten and nearly eight times respectively; whilst at sixty-five and upwards the rate for males was nearly three times and that for females more than twice as high among the insane as in the population at large.

There is not much variation from year to year in the proportions of the sexes amongst the certified insane. On January 1, 1910, for every thousand persons there were 463.6 males and 536.4 females; a year previously there were 463 males to 537 females. Since 1859 there has been, however, a gradual tendency for the proportion of males to increase, the relative proportions in that year being males 455.8, females 544.2.

The figures which refer to the distribution of those directly admitted into institutions of the insane in the three categories of "Single," "Married," and "Widowed," yield almost precisely the same results as those dealt with in the last report. At marriageable ages, twenty-five and upwards, the proportion of unmarried of both sexes is considerably higher amongst the insane than it is in the general population, and to a slighter extent, also, is the proportion of the widowed. But whereas the married in the population at large at these age periods are to the whole number of males 72 per cent., females 64 per cent., the proportions amongst those admitted are males 52 and females 48. In respect to the total population of each class the calculations show that at the marriageable ages considerably more single than married or widowed persons were admitted to care.

Of all the patients annually admitted into institutions for the insane, concerning whom reliable information has been obtained, and excluding cases of congenital insanity, about three-fourths were admitted for their first attack of insanity, a rather larger proportion than this obtaining amongst the males and a smaller amongst females. Those received for the first time into care amounted to 82.6 per cent. of the average total admissions for the two years, and, therefore, 17.4 per cent. had previously suffered from mental disorder without recourse to institutional treatment. But 26.7 per cent. were when admitted not suffering for the first time, and therefore 9.3 per cent. in their early attacks must have been kept at home or detained in workhouses or elsewhere. It is thus apparent that at present sufficient information does not exist to give an accurate estimate of prevailing insanity.

In a lengthy table details are given of the yearly average number of direct admissions of patients during the two years 1907 and 1908, classified according to their occupations. The Commissioners, however, prefer to wait until the figures for a longer term are available for comparison before commenting on them.

The report sets forth, by means of tables, the assigned causes of insanity. Of the 50 antecedents which constitute the list there are 12 furnishing between them two-thirds of the total number of instances, among which the factor considered to be the principal or most effective in inducing the mental disorder was (in order of comparative frequency) the following: (1) The puerperal state, which in 82 per cent. of the number of instances recorded of it was regarded as the principal cause; (2) epilepsy; (3) sudden mental stress—*i.e.* violent shock or emotion; (4) alcoholism (personal); (5) influenza, which, although not numerically frequent, was considered to be the most manifest cause in the attack in 67 per cent. of the instances recorded of it; (6) prolonged mental stress; (7) senility, which often stands alone as the determining cause of dementia; (8) the climacteric in women, well recognised as a period marked by nervous perturbation; (9) congenital mental defect; (10) an insane heredity, perhaps the most potent factor predisposing to insanity in an individual; (11) injuries; and (12) syphilis, which in 50 per cent. of its total occurrences was regarded as the principal agent. These ætiological factors may, therefore, be regarded as probably having the chiefest influence in the production of insanity.—"Sixty-fourth Report of the Commissioners in Lunacy" (204). Price 2s. 11d.

